# Factors Contributing to Self-Medication and Consumption of Non-Prescribed Drugs in Portugal

**DOI:** 10.3389/ijph.2022.1604852

**Published:** 2022-11-07

**Authors:** Aida Isabel Tavares, Pedro Lopes Ferreira, Veronica Cavadas

**Affiliations:** ^1^ CEISUC - Centre for Health Studies and Research, University of Coimbra, Coimbra, Portugal; ^2^ ISEG, UL - Lisbon School of Economics and Management, University of Lisbon, Lisbon, Portugal; ^3^ FEUC - Faculty of Economics, University of Coimbra, Coimbra, Portugal; ^4^ USF Marquês de Marialva, ACES Baixo Mondego, Coimbra, Portugal

**Keywords:** predictors, non-prescribed drugs, OTC, medical care, Portugal

## Abstract

**Objectives:** This work sets out to find the relationship between taking non-prescribed drugs and predisposing, enabling and need factors. Specifically, our main aim is to find the relationship between taking non-prescribed drugs and the lack of health care.

**Methods:** We used data from the last 2019 National Health Survey and estimate logistic regressions for the whole sample and stratified by sex.

**Results:** The most striking finding is that people self-medicating with non-prescribed drugs seem to be replacing health care when this is not used because of financial constraints or distance from provider. This suggests that non-prescribed drugs are a fast, affordable, alternative to health care. Other findings show that income and the financial resources to cope with unexpected expenditure are considerations in taking these drugs. Health and needs are other factors triggering their consumption.

**Conclusion:** Policy measures need to be aimed at improving access to medical care, providing responses to health needs such as those arising from chronic pain, and improving health literacy.

## Highlights

1) People self-medicating with OTC seem to be substituting health care because of financial and distance constraints.
2) Income and the financial capacity to cope with unexpected expenditure are associated with taking OTC drugs.
3) Sleeping problems, chronic pain, and using an emergency contraceptive pill are drivers for taking OTC drugs.

## Introduction

A strategic change in the pharmaceutical market that has occurred in recent years is the switch from prescription drugs (Rx) to over-the-counter (OTC) drugs (1). Additionally, the OTC market has been liberalized (in Portugal since 2005) meaning that OTC drugs can be sold by retailers as well as by community pharmacies. These changes have been followed by another social and medical change, namely the promotion of self-medication (SM). This is the selection and use of drugs by an individual to treat self-recognized illnesses or symptoms, without consulting a doctor (2, 3). These symptoms include pain, allergies, migraine, skin conditions, fungal infections, cold symptoms, fever control, heartburn, and dyspepsia (4). However, the practice of SM not only includes the use of OTC but also Rx drugs which people may acquire improperly, without prescription (3). In our work, we focus on OTC, or also referred as nonprescribed drugs, and disregard drugs requiring prescription such as antibiotics, that is, our focus is on drugs which can be bought without prescription. Some of these OTC may be prescribed, such as vitamin D, but this alternative is not covered because this purchase is based on a prescription.

SM with OTC consumption has several advantages. On the one hand it saves the opportunity cost of the time which physicians spend taking care of minor illnesses, and at the same time it saves people having to take time to go to medical appointments, as well as the possible payment for the appointment (5). On the other hand, it gives people power over their health and so it has benefits for their labour productivity (6). Despite these well-recognized advantages, SM poses risks and disadvantages for peoples’ health. These include making a wrong diagnosis, taking the wrong amount of a drug, polypharmacy, adverse reactions and drug interactions, addiction, abuse and overdose, and also delaying correct diagnosis by masking other severe health conditions (4, 5).

In Portugal, the OTC market represents about a quarter of the whole pharmaceutical market (volume), while a quarter of the OTC market involves analgesics and antipyretics, and about 15% to topical application (7). OTC drugs are sold in community pharmacies and other authorized retailers, such as drug outlets and supermarkets; advertising OTC is possible under certain conditions and rules, and buyers must be older than 16 years of age (8). The Portuguese health system is defined as a Beveridge-type national health service, and it is facing some well-identified challenges (9, 10) such as waiting lists, unreasonable time to get an appointment, unavailability of general practitioners, unmet health needs, and high cost-sharing of prescribed pharmaceuticals.

Given this contextual and institutional framework that characterizes the country’s health system, the aim of our analysis is to find the factors associated with self-medication and OTC consumption. More specifically, we aim to test the possible substitutive relationship between the lack of health care and OTC provision, and to understand the role of individual health needs and health in the consumption of OTC drugs in Portugal.

**FIGURE 1:**
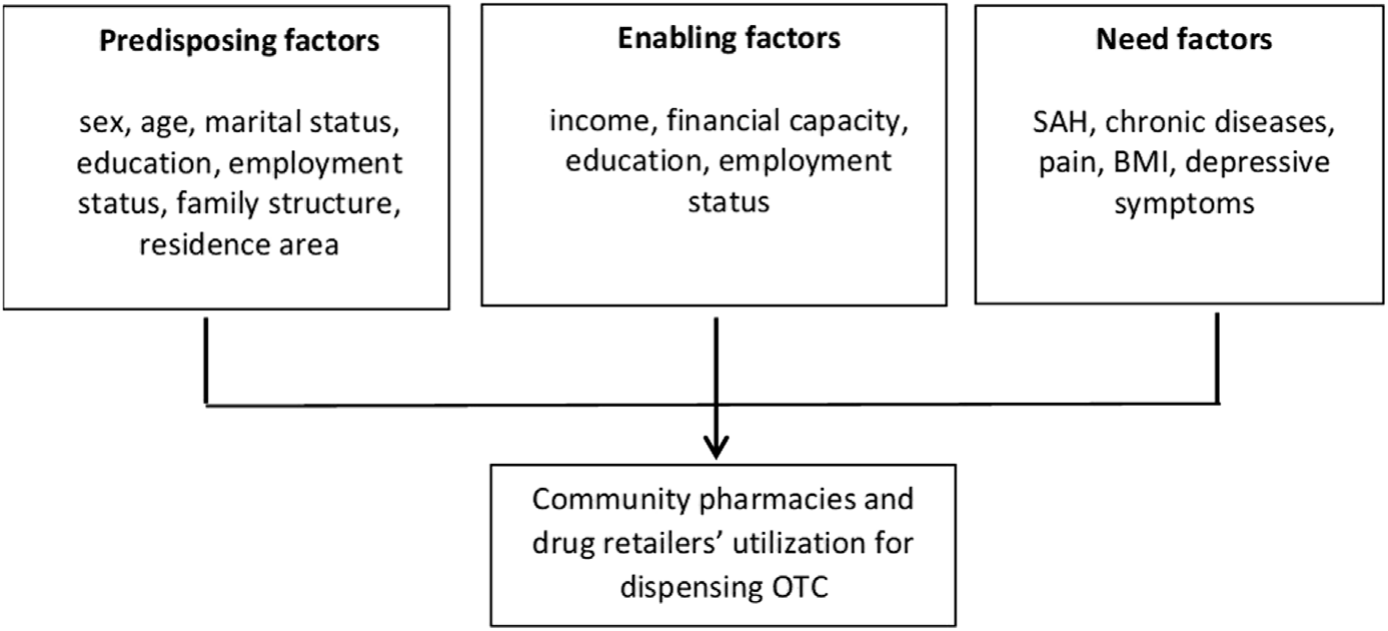
Conceptual framework of Anderson’s Behavioral Model of Health Services Use (Portugal, 2022).

The conceptual model that sustains our analysis is the Behavioral Model of Health Services Use suggested by R. Andersen in 1968 (11). Since then, the model has been updated and adjusted (12), and used to support different empirical studies, including those related to self-care (13–18). Andersen’s model includes three major factors that influence the use of health services at both the individual and contextual levels, namely, 1) predisposing, 2) enabling, and 3) need factors (11, 12, 14) as shown in Figure 1. Predisposing factors mainly include demographic and community characteristics; enabling factors are related to the financing and organizational characteristics which are conditions for enabling and facilitating the use of pharmacies and other shops to sell medicaments OTC; needs are the factors that result in the action to self-medicate and to consume OTC medicines, which are related to the type and duration of illness or symptoms.

A large number of studies on self-medication are focused on low-to-medium-income countries and few of them are aimed at high-income countries (19). Empirical evidence concerning demographic, economic and medical care factors as drivers for OTC use or self-medication in Europe is diversified but some results are aligned. Whereas in Eastern European countries, Austria and in the UK, men tend to self-medicate more often than women (20–22), the opposite is true in Spain (23, 24), and it has no statistical significance in Portugal (25). We find that older people are more likely to take non-prescribed drugs in countries such as Bulgaria, Slovakia and the UK (20, 22), while in others, like Spain (24) and Austria (21), such tendency is not found. In general, better education and higher income are associated with a greater probability of taking OTC drugs (20–25).

This type of concern is more often found in studies for low-to-medium-income countries (19). The influence of medical care on SM has been given little attention in European countries because of the universal coverage of health systems. In Spain and Serbia (24, 26) findings point to a negative relationship between medical consultation and self-medication, but in Austria (21) the opposite happens, and in Portugal (25) no statistical significance is found. From a worldwide perspective (19), difficult access to medical care is an important driver of self-medication.

The importance of health and health needs in SM has captured more attention in European studies. The findings point towards a general association between lower health status and chronic diseases and OTC consumption (20–22).

## Methods

### Data and Sample

The data used in this analysis were collected by the 2019 National Health Survey. The National Health Survey applied a sampling method based on stratification and multistage data collection to ensure that it is representative of the Portuguese population (27). Data were collected between September 2019 and January 2020. This survey is standardized and regulated at European level (European Parliament and European Council Regulation no 1338/2008; European Commission Regulation no 2018/255). It includes 14,617 non-institutionalized individuals older than 15.

Our sample considers respondents aged over 18, so it includes 14,275 individuals. We excluded 276 minors aged between 15 and 17, because not only do they not have complete autonomy in deciding on the consumption of OTC drugs, but they usually do not have the financial autonomy to buy those drugs either (unless provided by parents). We also excluded 66 pregnant women (of whom only 11 took OTC medicines) because it was assumed that they are only supposed to take OTC under medical supervision.

### Outcome Variable

The outcome variable measures the decision to self-medicate and to consume OTC medicines. This variable is obtained from the following question: “In the last 2 weeks, did you take any drug, natural medicine or vitamins which were not prescribed by a physician? (Do not include birth control pills or other birth control hormones).” The answer is binary, meaning, “yes” or “no”. This variable is labelled as “OTC”.

### Independent Variables

The set of independent variables were divided into three groups: predisposing, enabling, and need according to Anderson’s Behavioral Model of Health Services Use. These variables are listed in Figure 1 and described in Table 1.

**TABLE 1:** Predisposing, enabling and needs factors—independent variables (Portugal, 2022).

Variables name	Description
Predisposing factors	
Male	Dummy variable. Takes the value 1 if male; 0 if female
Age	The values of mid-point of the age group age of the class of age
Education	The number of years of completed school
No_family	The number of household members
Civil status	
Single	Dummy variable. Takes the value 1 if single; 0 otherwise
Married	Dummy variable. Takes the value 1 if married; 0 otherwise
Widow/widower	Dummy variable. Takes the value 1 if widow/widower; 0 otherwise
Divorced	Reference category
Residence area	Additional information in Supplementary Material SM1
Urban	Dummy variable. Takes the value 1 if individual lives in an urban area; 0 otherwise
Rural	Dummy variable. Takes the value 1 if individual lives in a rural area; 0 otherwise
Moderate urban	Reference category
Enabling factors	
Income (quintiles)	
Q2, Q3, Q4 and Q5	Dummy variables. Take the value 1 if income is in each quintile; 0 otherwise
Q1 (poorest)	Reference category
Employment_status	
Employed	Dummy variable. Takes the value 1 if employed; 0 otherwise
Unemployed	Dummy variable. Takes the value 1 if unemployed; 0 otherwise
Student	Dummy variable. Takes the value 1 if student; 0 otherwise
Retired	Dummy variable. Takes the value 1 if retired; 0 otherwise
All remaining status	Reference category. This residual category includes domestic work, community work, unable to work and other situations of labour inactivity
Financial_availability	Dummy variable. Takes the value 1 if the household has financial availability to pay unexpected health expenses without asking for a loan; 0 otherwise
Need factors	
Unmet healthcare needs	Additional information in Supplementary Material SM2
Unmet_needs	Dummy variable. Takes the value 1 if unmet health needs (appointment or treatment) due to financial difficulties in the last 12 months are reported; 0 otherwise
Waiting_list	Dummy variable. Takes the value 1 if respondent has been waiting for a medical appointment, examination or treatment for the last 12 months beyond the reasonable waiting time; 0 otherwise
Waiting_distance	Dummy variable. Takes the value 1 if respondent has been waiting for a medical appointment, examination or treatment for the last 12 months due to distance or lack of transportation; 0 otherwise
Health and needs	
SAH	Self-Assessed Health. Takes values between 1 and 5, where 1 is the worst level and 5 the best
Chronic	Dummy variable. Takes the value 1 if respondent has at least one chronic or long-standing illness; 0 otherwise
Pain intensity	Takes values between 1 and 6, where 1 = no pain and 6 = very intense pain
Lack sleep	Dummy variable. Takes the value 1 if respondent reports lack of sleep or difficulty sleeping; 0 otherwise
Lack courage	Dummy variable. Takes the value 1 if respondent reports feeling depressive or sad; 0 otherwise
Lack energy	Dummy variable. Takes the value 1 if respondent reports lack of energy or feeling fatigue; 0 otherwise
Lack focus	Dummy variable. Takes the value 1 if respondent reports failure or difficulty in focusing or concentrating; 0 otherwise
Depression score	This score is based on the EURO-D scale and it is computed as the sum of EURO-D available in the survey. Additional information in Supplementary Material SM3
Other needs	
BMI	Body Mass Index. This indicator is calculated as the ratio between weight in kilograms and the square of height in meters
Birth_control_pill	Dummy variable. Takes the value 1 if women report taking a birth control pill; 0 otherwise
Emergency_pill	Dummy variable. Takes the value 1 if women report ever having taken an emergency contraceptive pill (next day pill) to prevent pregnancy; 0 otherwise

Some points about some of the independent variables are worth mentioning.

Firstly, looking at the variable of age, which is collected and provided in age classes, we considered the mid-point value of the age class because when using the factorial variables for age class, the level of multicollinearity exceeds the accepted level for the value of inflated factor (VIF test). The value for the oldest class is taken to be 85 which is the floor value of the age interval.

Secondly, although the question about whether the respondent took OTC medicines clearly excludes the birth control pill and other women’s hormones, we tested a possible relationship between the responses to OTC questions and the responses given by women taking the pill and the emergency contraceptive pill. We consider taking contraceptives as a need to prevent pregnancy. Women taking the pill need to go to the pharmacy more often and those who take the emergency contraceptive pill have a learning experience on accessing OTC drugs in community pharmacies. In this way, women may be more willing to demand OTC, either due to a more frequent relationship with the pharmacist or due to more frequent opportunity to buy drugs.

Thirdly, we have considered BMI in the ‘need factors’ group because high levels of BMI, either measurable or perceived by people, may create an urge to reduce weight, which may motivate a demand for weight-reducing drugs.

Additionally, we have considered other perceived needs which may trigger individuals to seek OTC drugs, needs such as, lack of sleep, lack of courage/well-being, lack of energy, and lack of focus, for which there are specific OTC remedies. Besides these needs, we have also computed a depression score based on EURO-D scale to measure depression in the individual. Our score (“depression_score”) is a sort of reduced EURO-D because it does not include all EURO-D items (28). For a more detailed explanation see Supplementary Material SM2.

### Quantitative Analysis

The analysis performed in this work is divided into two main sections. In the first section we present the results obtained from a descriptive statistic.

Next, we estimate a logistic regression for all the sample, for each sex and also for the people aged less than 80. First, we separate women and men because men do not buy or take birth control pills and there are sex differences in the access and utilization of preventive health care services (29). So, we take sex differences as given by previous empirical work and we do not aim to test sex differences (30, 31). Second, we exclude very old seniors (that is, older than 80) to prevent any kind of age bias. We consider an estimation including ‘depression score’ instead of single needs related to mental health.

A VIF test is performed to test multicollinearity across independent variables. The *linktest* is used for testing specification errors and the Hosmer-Lemeshow is applied for goodness-of-fit test. The reported standard errors are robust to mitigate any heteroscedasticity. Finally, the results are reported using odds-ratios (OR), and 95% confidence intervals (CI) and p-values are also displayed. All the analyses presented in this work are performed in STATA 16.

## Results

### Descriptive Analysis

The number of people acknowledging that they took non-prescribed drugs was 3,208, which accounts for about 22.5% of our sample of 14,275 people. Of these, most are women, about 63%.

A statistical description of the set of independent variables is presented in Table 2. Of these, we would like to highlight the statistics showing the lack of health care that people report. More than 4% of people indicate waiting for an appointment, treatment, or medical exam due to long distance or lack of transport as factors; nearly 25% of people report waiting for health care beyond the reasonable time, meaning that they are on a waiting list for too long, and about 10% of people report being unable to cope with their health needs due to financial difficulties.

**TABLE 2:** Statistical description of independent variables (Portugal, 2022).

Variables	Statistical description: Prevalence as % of sample (absolute frequency); mean values; other information
Predisposing factors	
Male	43.4% (6,195) of individuals are males; 65.6% (8,080) are females
Age	The mean age is 56.7 years; additional information in Supplementary Material SM4
Education	The average number of years of schooling is 12; 42% of people have 12 years of schooling or less; additional information in Supplementary Material SM5
No_family	The average number of household members is 2. This variable ranges from 1 to 7
Civil status	
Single	23.9% (3,418)
Married	50.1% (7,147)
Widow/widower	15.6% (2,231)
Divorced	10.1% (1,434)
Residence area	
Urban	28.9% (4,130)
Rural	32.7% (4,673)
Moderate urban	38.3% (5,472)
Enabling factors	
Income (quintiles)	
Q1 (poorest)	18.3% (2,615)
Q2	24.7% (3,531)
Q3	20.7% (2,961)
Q4	18.1% (2,576)
Q5 (richest)	18.2% (2,592)
Employment_status	
Employed	45.0% (6,418)
Unemployed	7.0% (995)
Student	3.0% (425)
Retired	36.1% (5,148)
All remaining status	9.1% (1,257)
Financial_availability	64.6% (8,844) have financial availability to pay unexpected health expenses; 35.4% (4,855) of respondents have no such capacity
Need factors	
Unmet needs	
Unmet_needs	10.0% (1,415)
Unmet_ waiting	24.8% (3,524)
Unmet_distance	4.6% (657)
Health and needs	
SAH	15.9% of individuals report lower levels of health status [levels 1 and 2]; 43.1% report higher level of health status [levels 4 and 5]; additional information in Supplementary Material SM6
Chronic disease	57.8% report suffering from some chronic or prolong disease
Pain intensity	37.0% of individuals report no pain and 16.4% report high levels of pain intensity [levels 5 and 6]; additional information in Supplementary Material SM7
Lack sleep	16.1% (2,291)
Lack courage	8.8% (1,250)
Lack energy	13.7% (1,960)
Lack focus	4.6% (651)
Depression_score	It is a EURO-D scale-based score; it varies between 1 and 28, where 1 means never and 28 means almost every day for a set of feelings related with mental health
Other needs	
BMI	The mean value is 26.4. The minimum value is 14.6 and maximum value is 54.4
Birth_control_pill	13.8% (1,111)
Emergency_pill	7.5% (606)
Sample size	14,275 [women: 8,080]

### Estimated Results

The results obtained from the VIF test for testing potential multicollinearity across independent variables is presented in Supplementary Appendix Table SA1. There is no evidence for multicollinearity. The pairwise correlations between non-binary variables are presented in Supplementary Appendix Table SA2.

The results for the estimated logistic regression are presented in Table 3, for the whole sample and for women and men separately.

**TABLE 3:** Logistic estimation results for over-the-counter drugs consumption (Portugal. 2022).

	ALL SAMPLE					MALES					FEMALES				
	OR		95%CI		*p* > z	OR		95%CI		*p* > z	OR		95%CI		*p* > z
Predisposing factors															
Male	0.750***		0.684	0.822	0.000										
Age	0.993***		0.988	0.997	0.003	0.995		0.988	1.003	0.234	0.993**		0.986	0.999	0.024
Education	1.013**		1.003	1.022	0.012	1.014		0.997	1.032	0.115	1.011*		1.000	1.023	0.060
Nr_family	0.968		0.925	1.014	0.173	0.961		0.893	1.035	0.297	0.972		0.916	1.033	0.361
Civil status															
Single	0.922		0.786	1.083	0.323	1.023		0.787	1.330	0.862	0.884		0.720	1.086	0.240
Married	0.886		0.763	1.028	0.110	0.909		0.709	1.166	0.452	0.908		0.751	1.098	0.319
Widow/widower	1.053		0.879	1.262	0.573	1.129		0.782	1.631	0.516	1.051		0.848	1.301	0.652
Divorced															
Residence area															
Urban	1.149***		1.039	1.272	0.007	1.148*		0.977	1.350	0.094	1.140**		1.001	1.299	0.048
Rural	0.893**		0.806	0.989	0.030	0.771***		0.655	0.909	0.002	0.987		0.866	1.126	0.850
Moderate urban															
Enabling factors															
Income															
Q1 (poorest)															
Q2	1.121		0.959	1.310	0.152	1.179		0.877	1.585	0.276	1.089		0.901	1.316	0.377
Q3	1.151*		0.977	1.355	0.092	1.074		0.801	1.440	0.633	1.217*		0.994	1.490	0.057
Q4	1.373***		1.166	1.617	0.000	1.289*		0.965	1.721	0.086	1.433***		1.169	1.758	0.001
Q5 (richest)	1.733***		1.463	2.052	0.000	1.630***		1.208	2.199	0.001	1.772***		1.437	2.185	0.000
Employment_status															
Employed	1.109		0.916	1.342	0.291	0.902		0.589	1.382	0.637	1.153		0.925	1.437	0.204
Unemployed	1.118		0.888	1.408	0.342	0.851		0.538	1.347	0.492	1.204		0.913	1.588	0.188
Student	1.216		0.882	1.677	0.233	0.982		0.557	1.733	0.951	1.394		0.924	2.103	0.114
Retired	0.986		0.813	1.197	0.889	0.771		0.497	1.194	0.244	1.066		0.853	1.331	0.575
All remaing status															
Financial_availability	1.128**		1.019	1.249	0.020	1.224**		1.030	1.456	0.022	1.079		0.951	1.225	0.238
Needs															
Health and needs															
SAH	1.064*		0.999	1.132	0.054	1.082		0.977	1.197	0.130	1.061		0.979	1.149	0.152
Chronic	1.011		0.911	1.121	0.843	0.982		0.831	1.162	0.834	1.031		0.902	1.180	0.653
Pain intensity	1.193***		1.156	1.232	0.000	1.225***		1.163	1.290	0.000	1.173***		1.127	1.221	0.000
Lack courage/well-being	0.905		0.754	1.088	0.288	0.869		0.596	1.266	0.464	0.914		0.741	1.127	0.400
Lack sleep	1.153**		1.012	1.313	0.032	1.340**		1.066	1.685	0.012	1.067		0.912	1.250	0.417
Lack energy	1.100		0.945	1.280	0.217	1.174		0.887	1.554	0.262	1.070		0.894	1.280	0.460
Lack focus	0.914		0.726	1.152	0.448	0.771		0.475	1.250	0.291	0.972		0.746	1.268	0.836
Unmet healthcare needs															
Unmet_needs	1.233***		1.066	1.427	0.005	1.211		0.942	1.557	0.134	1.261***		1.054	1.509	0.011
Waiting_list	0.724***		0.583	0.899	0.003	0.798		0.544	1.171	0.249	0.688***		0.529	0.894	0.005
Waiting_distance	1.263***		1.143	1.397	0.000	1.276***		1.078	1.510	0.005	1.246***		1.099	1.412	0.001
Other Needs															
BMI	0.986***		0.977	0.996	0.006	0.982**		0.964	0.999	0.039	0.990*		0.979	1.002	0.095
Emergency_pill											1.348***		1.105	1.643	0.003
Birthcontrol_pill											**0.889**		0.747	1.058	0.184
Constant	0.219***		0.127	0.378	0.000	0.173***		0.070	0.424	0.000	0.201***		0.098	0.412	0.000
Number of obs	13,155					5740					7388				
Wald chi2 (29)	466.86					192.52					235.28				
Prob > chi2	0.0000					0.0000					0.0000				
Pseudo R2	0.0344					0.0360					0.0290				
Log pseudolikelihood	−6,807.220					−2,711.719					−4,065.832				
Linktest															
_hatsq (P > z)	0.0062				0.935	0.156				0.145	0.025				0.824
Hosmer-Lemeshow chi2 (Prob > chi2)	3.71				0.8819	8.13				0.4212	7.55				0.4781

Note: _cons estimates baseline odds; ***p < 0.01; **p < 0.05; *p < 0.1. Bold indicates statistically significant OR.

We begin by presenting the results related to need factors, specifically the unmet healthcare needs. The most relevant results show there is a significant correlation between taking OTC medicines and (the lack of) health care. There seems to be a substitution effect between OTC consumption and both unmet health care needs due to financial constraint, and waiting for health care due to long distance; the relationship between waiting for health care on a waiting list has a complementary effect. In other words, people who report unmet healthcare needs because of financial difficulties or experience lack of health care due to distance are more likely to report taking OTC remedies than those who do not report those deficits in health care by a odds ratio (OR) equal to about 1.2.

On the other hand, people reporting waiting for medical care for longer than a reasonable time are less likely to report taking OTC drugs by a OR equal to 0.724. These relationships are found in women, but they are not statistically significant in the estimation for men, except for the case of waiting for health care due to long distance or lack of transport.

Regarding the influence of health and needs on OTC consumption, we found that painkillers are common OTC drugs because of the increasing likelihood of OTC purchases associated with pain intensity, by a OR of 1.193. We also found that the need to sleep motivates a demand for OTC drugs, although this tendency was not found significant for women. We tested the model to include the depression score and we found a positive association between this score and taking OTC medicines (results presented in Supplementary Material SM8), meaning that demand for OTC increases with the number of depression symptoms (OR is equal to 1.036 for *p-value* less than 0.0001).

Regarding the relevance of other needs, our results show that higher BMI is associated with a lower likelihood of taking OTC products, despite the estimated factor being very close to value 1. In the case of OTC drugs taken by women, we found that women taking an emergency birth-control pill are more likely to take an OTC drug by a OR of 1.348 than women who do not use this type of pill.

With respect to enabling factors, as expected people reporting higher incomes or financial ability to deal with unexpected health expenses are more likely to report buying OTC, except for women, for whom financial ability has no statistical significance.

With reference to the predisposing factors, we found that men and older people are less likely to buy drugs OTC; people with higher levels of education are more likely to choose OTC remedies. Finally, those who live in urban areas are more likely to buy OTC products while those who live in rural areas are less likely to do so than those living in moderate urban areas.

Closing this section, the model specification test (link test) and goodness-of-fit test (Hosmer-Lemeshow) show that the model has a high goodness-of-fit and is well specified. We also tested the model using a sample including people younger than 80 and the results are identical and in line with those presented above (results displayed in Supplementary Material SM8).

## Discussion

Self-medication by consuming OTC drugs is a widespread phenomenon in Europe as more Rx drugs are shifted into OTC providers and people are increasingly empowered over their health. There is little empirical evidence about the factors associated with taking non-prescribed drugs in different countries in Europe. Portugal has a specific institutional structure which is characterized by a national health service with universal coverage, but small cost sharing for prescribed pharmaceuticals, and OTC medicines are easily sold in pharmacies and authorized retailers. We aimed to find the predisposing, enabling and need factors associated with the consumption of non-prescription drugs and to test its relationship with the lack of health care.

### Key Findings

The most relevant result concerns, first, a substitutive relationship between taking OTC and facing healthcare needs that are not met because of financial constraints and long distance and/or lack of transport. Secondly, we found a complementary relationship between taking OTC remedies and experiencing unmet health care arising from being placed on a waiting list.

Other results show the importance of other need factors, such as pain intensity, lack of sleep, and pregnancy concerns. Additionally, some predisposing and enabling factors may also play a role in an OTC purchasing decision.

### Interpretation

Firstly, the main findings concern the correlation between the consumption of OTC drugs and unmet health care needs. We found a substitution relationship between OTC drugs and unmet healthcare needs due to financial difficulties or long distance, and a complementary relationship between OTC drugs and unmet health care due to waiting lists.

These findings show that people may choose to take OTC drugs to replace medical care due to financial and distance/transport constraints, which may indicate that OTC remedies are an affordable and faster alternative for health care. This result has been found before in low-medium income countries and those with weak social welfare (19), and it certainly raises concerns for policymakers and health professionals. Delaying medical care is a serious option as it delays diagnosis and worsens health conditions, which should be prevented. A less expected finding, but one which raises less concern, is the correlation between consuming OTC products and the waiting time for medical care beyond what is reasonable. People seem to stay on hold, expecting a medical assessment of their problem, waiting for a medical intervention or diagnosis. There may be some fear about masking illness conditions, or even speeding up illness. Waiting for a medical appointment is the intrinsic hope for improving health.

Second, regarding the drivers related to health and needs some general results were found in the literature (21, 22, 24, 25) but we have not been able to fully acknowledge. Pain intensity and lack of sleep motivate people to consume OTC drugs according to our results, as well as better reported health. Painkillers are the main class of non-prescribed drugs sold in the OTC market, so this significant result could be expected. This consumption of painkillers might raise concern for potential adverse effects arising from excess consumption, polypharmacy, and pain interactions (32). On the other hand, Portugal faces a mental health problem and it is the European country where most antidepressants are sold (33, 34). So perhaps this high prevalence of mental health issues could be reflected in a high demand for OTC drugs, specifically, for drugs to help with sleep. And in fact, this mental health problem is also observable in the positive correlation found between depression score and OTC demand.

Other needs have also been found to play a part in taking OTC remedies. Women taking emergency contraceptive pills are more likely to self-medicate with OTC drugs. Emergency contraceptive pills can be obtained either with a medical prescription or in a community pharmacy after a licensed pharmacist’s recommendation. Easier access means it is very likely that most women got their emergency pills from a pharmacy. This emergency provides women with a learning experience on accessing and using community pharmacy services, including buying OTC drugs (35). This association has not been found in previous studies, but it may merit future research about the proximity between people and pharmacists within health care.

Third, predisposing factors for taking OTC drugs found in this analysis are being female, young, having education, and living in urban areas. First, men tend to take less care of their health and do so much later than women (23, 33, 36, 37). Second, education is associated with higher individual resources (cognitive, communicative, informative, relational) and so people use their knowledge to exert their empowerment over health (20, 21, 23). Third, younger individuals tend to use more OTC medicines (38), most likely because their opportunity cost for looking for health care is higher and because the illness is not serious enough to motivate them to seek out health care. However, this result may differ, according to different views and analyses (39). Finally, pharmacies and other retailers selling drugs are less available in rural areas, so a lower OTC consumption is quite likely. However, this difference can raise concerns of equity of access to drugs, not only OTC ones, but prescribed drugs too. The greater the distance to a pharmacy means it is accessed less often, and so in cases of mild illness, people may choose to put up with feeling unwell.

Fourthly, significant enabling factors for OTC consumption are in fact of an economic nature. People with higher incomes or with financial resources to deal with unexpected health expenses are more likely to choose OTC products, as suggested by other empirical evidence (12, 21). If one regards an OTC product like any other good in the market, then there is no reason for concern; however, if one believes that an OTC product has relevance for living well, leading a normal life, and being productive in society, then these economic differences may reflect a socioeconomic gradient (40).

Lastly, the statistically significance differences found between women and men for the variables age, education, urban, financial availability, lack of sleep, unmet needs and waiting lists may be explained by several reasons: 1) the size of the men sample is smaller, 2) women and men differ in the expression of health needs, in the use and access to health care, and 3) women have more frequent contact with pharmacist than men due to the birth control need. Nevertheless, for these variables, the direction of the association is the same between men and women.

### Strengths and Limitations

There are some limitations to our analysis which should be noted. First, the question concerning self-medication asks people if they took any drug which was not prescribed by a physician. However, people can interpret this question in differently. They might take drugs that were in the bathroom cabinet but were prescribed long before, or take drugs which were prescribed for a family member, neighbour or friend. Nevertheless, the framing of this question in the survey was considered carefully. It comes after a question aimed at prescribed drugs. This will tend to eliminate prescribed drugs from the mind of the respondent in the next question about OTC drugs.

Second, the question about non-prescribed drugs refers to 2 weeks before. The main reason for this time consideration, apart from being general European reference, could be for reasons related to people’s memory. This could be slightly biased, but we believe that this sampling bias is not significant because no matter the time of the year, there are always conditions calling for the consumption of OTC drugs. In the winter people will tend to take flu-related drugs and in the spring/summer people will be more likely to take allergy-related drugs.

Another possible limitation concerns the cross-section analysis which, despite not allowing for causality analysis, nonetheless provides good clues on the associated factors motivating the decision to consume OTC drugs.

The major strength of our work is twofold. Not only does it use recent data for Portugal, which fit with a specific institutional setting for the health system, but it also contributes to the understanding and comparison of the profile of people buying OTC drugs in high-income countries in Europe. This work also provides results with relevant potential policy implications.

### Policy Implications

From a policy perspective, our work has brought important insights related to the drivers of self-medication with non-prescribed drugs. First, the substitution effect between consuming OTC drugs and health care due to financial or distance/transport difficulties calls for measures to mitigate these difficulties. Not only for equity reasons, but also for health reasons. People may be delaying medical care and masking symptoms which will become more severe or delay a serious diagnosis, so improving access to medical care is an important policy measure. The widespread trend of health digitalization could be used to decrease distance and transport strains.

Second, pain management and primary care needs to be considered when designing functions of primary care units. Chronic pain impacts peoples’ lives in several ways and it can also lead to mental health problems. General physicians often under-treat pain and are also uncomfortable treating pain because of the various health complexities related to it. However, training or finding specialists in pain management can help to improve people’s quality of life and contribute to a balanced consumption of painkillers.

Third, mental healthcare support has long been recognized as an important policy measure that is still lagging behind. Another related policy is the need to improve citizens’ health literacy so that they are better able to navigate the health system and manage their own health. Taking OTC drugs has advantages and disadvantages, and when people are aware of this trade-off the risks associated with OTC consumption could then be reduced.
