# Supplementary material for: Factors Contributing to Self-Medication and Consumption of Non-Prescribed Drugs in Portugal
Source: Int J Public Health. 2022 Nov 7;67:1604852. doi: 10.3389/ijph.2022.1604852 (PMC9676243; doi:10.3389/ijph.2022.1604852)
Supplement: Supplementary file 1 [file Table1.docx]

**APPENDIX**

“Table A1: VIF test (Portugal. 2022)”

| **Variable** | **VIF** | **1/VIF** |
| --- | --- | --- |
| male | 1.34 | 0.743885 |
| urban | 1.27 | 0.785618 |
| rural | 1.28 | 0.778474 |
| age | 4.29 | 0.232870 |
| single | 2.86 | 0.349996 |
| married | 3.37 | 0.296418 |
| widow | 2.52 | 0.396636 |
| education | 1.16 | 0.862261 |
| income |  |  |
| Q2 | 2.47 | 0.404485 |
| Q3 | 2.44 | 0.409046 |
| Q4 | 2.30 | 0.434824 |
| Q5 | 2.55 | 0.392146 |
| financial_availability | 1.29 | 0.774651 |
| nr_family | 1.60 | 0.624862 |
| student | 1.74 | 0.575827 |
| employed | 5.17 | 0.193557 |
| unemployed | 1.97 | 0.507542 |
| retired | 4.80 | 0.208169 |
| BMI | 1.09 | 0.921606 |
| SAH | 2.07 | 0.482336 |
| chronic | 1.48 | 0.673708 |
| pain intensity | 1.51 | 0.661125 |
| lack courage/well-being | 1.55 | 0.646998 |
| lack sleep | 1.37 | 0.727635 |
| lack energy | 1.66 | 0.601538 |
| lack focus | 1.30 | 0.772113 |
| waiting_distance | 1.17 | 0.853064 |
| waiting_list | 1.12 | 0.889904 |
| unmet_needs | 1.16 | 0.858867 |
| birth_control_pill | 1.25 | 0.796952 |
| emergency_pill | 1.12 | 0.890449 |
| Mean VIF | 2.01 |  |

“Table A2: Pairwise correlations across non-binary variables (Portugal. 2022)”

|  | age | education | pain intensity | nr family | BMI | SAH |
| --- | --- | --- | --- | --- | --- | --- |
| age | 1.00 |  |  |  |  |  |
| education | -0.204 | 1.00 |  |  |  |  |
|  | 0.00 |  |  |  |  |  |
| pain intensity | 0.283 | -0.105 | 1.00 |  |  |  |
|  | 0.00 | 0.00 |  |  |  |  |
| nr family | -0.445 | 0.101 | -0.139 | 1.00 |  |  |
|  | 0.00 | 0.00 | 0.00 |  |  |  |
| BMI | 0.178 | -0.031 | 0.137 | -0.042 | 1.00 |  |
|  | 0.00 | 0.00 | 0.00 | 0.00 |  |  |
| SAH | -0.510 | 0.194 | -0.497 | 0.239 | -0.200 | 1.00 |
|  | 0.00 | 0.00 | 0.00 | 0.00 | 0.00 |  |
