# Supplementary material for: Factors Contributing to Self-Medication and Consumption of Non-Prescribed Drugs in Portugal
Source: Int J Public Health. 2022 Nov 7;67:1604852. doi: 10.3389/ijph.2022.1604852 (PMC9676243; doi:10.3389/ijph.2022.1604852)
Supplement: Supplementary file 2 [file DataSheet1.docx]

**Supplementary Material**

“SM1: Definition residence area (Portugal. 2022)”

Residence area is defined by the degree of urbanization. According to the criterion specified by Statistics Portugal/INE, an urban area is characterized as a geographical area with more than 5,000 inhabitants and a population density higher than 500 inhabitants per km^2^, and a rural area is defined as having fewer than 2,000 inhabitants and a population density below 100 inhabitants per km^2^.

“SM2: Questions measuring unmet health care needs (Portugal. 2022)”

The questions available in the National Health Survey measuring unmet health care needs are:

a) Did you had an appointment or medical treatment unmet due to financial difficulties in the last 12 months? [unmet_needs];

b) Are you waiting for an appointment, treatment or medical exam beyond the reasonable time in the last 12 months? [unmet_ waiting];

c) Are you waiting for an appointment, treatment or medical exam due to distance or transport in the last 12 months? [unmet_distance].

“SM3: Items included in the computation of depression score (Portugal. 2022)”

The EURO-D score items available in National Health Survey are depression, interest, sleep, appetite, fatigue, concentration, and guilt. The alternative answers are 1. Never; 2.Some days; 3.More the half of the days; 4.Almost every day. Depression score results from the sum of the ordered answers such that 1 means never and 28 means almost every day. The value 28 results from people answering almost every day in the 7 items.

“SM4: Statistical description of age (Portugal. 2022)”

| age interval | age  (mid-point) | freq. | % |
| --- | --- | --- | --- |
| 18-19 | 18.5 | 227 | 1.59 |
| 20-24 | 22 | 524 | 3.67 |
| 25-29 | 27 | 490 | 3.43 |
| 30-34 | 32 | 585 | 4.10 |
| 35-39 | 37 | 841 | 5.89 |
| 40-44 | 42 | 1,14 | 7.99 |
| 45-49 | 47 | 1,148 | 8.04 |
| 50-54 | 52 | 1,206 | 8.45 |
| 55-59 | 57 | 1,352 | 9.47 |
| 60-64 | 62 | 1,416 | 9.92 |
| 65-69 | 67 | 1,387 | 9.72 |
| 70-74 | 72 | 1,333 | 9.34 |
| 75-79 | 77 | 1,098 | 7.69 |
| 80-84 | 82 | 867 | 6.07 |
| +85 | 85 | 661 | 4.63 |

“SM5: Statistical description of education (Portugal. 2022)”

| education  (years) | Freq. | % |
| --- | --- | --- |
| 0 | 1,501 | 10.51 |
| 9 | 2,180 | 15.27 |
| 12 | 2,370 | 16.60 |
| 13 | 235 | 1.65 |
| 15 | 6,396 | 44.81 |
| 17 | 1,520 | 10.65 |
| 21 | 73 | 0.51 |
|  |  |  |

“SM6: Statistical description of self-assessed health (Portugal. 2022)”

| SAH level | Freq. | Percent |
| --- | --- | --- |
| worst level=1 | 544 | 3.82 |
| 2 | 1,732 | 12.15 |
| 3 | 5,835 | 40.94 |
| 4 | 4,746 | 33.30 |
| best level=5 | 1,394 | 9.78 |

“SM7: Statistical description of pain intensity (Portugal. 2022)”

| Pain intensity level | Freq. | Percent |
| --- | --- | --- |
| no pain =1 | 5,261 | 37.03 |
| 2 | 1,676 | 11.80 |
| 3 | 2,353 | 16.56 |
| 4 | 2,585 | 18.20 |
| 5 | 1,750 | 12.32 |
| strongest intensity =6 | 581 | 4.09 |

“SM8: Supplementary results (Portugal. 2022)”

|  | **Sample (age**≤**80)** | | | | |  | **All sample and depression score** | | | | | | | | | | | |
| --- | --- | --- | --- | --- | --- | --- | --- | --- | --- | --- | --- | --- | --- | --- | --- | --- | --- | --- |
|  | OR | P>z | 95% IC | | |  | OR | P>z | | | 95% IC | | | | |  |  |  |
| **Predisposing factors** |  |  |  |  |  | |  | | |  | | |  | |  | | |  |
| male | **0.744** | 0.000 | 0.676; | 0.818 |  | | **0.763** | | | 0.000 | | | 0.695; | | 0.836 | | |  |
| age | **0.994** | 0.030 | 0.989; | 0.999 |  | | **0.993** | | | 0.006 | | | 0.989; | | 0.998 | | |  |
| education | **1.014** | 0.019 | 1.002; | 1.025 |  | | **1.013** | | | 0.009 | | | 1.003; | | 1.023 | | |  |
| nr_family | 0.972 | 0.245 | 0.927; | 1.020 |  | | 0.969 | | | 0.185 | | | 0.925; | | 1.015 | | |  |
| civil status |  |  |  |  |  | |  | | |  | | |  | |  | | |  |
| single | 0.942 | 0.476 | 0.800; | 1.110 |  | | 0.924 | | | 0.334 | | | 0.787; | | 1.085 | | |  |
| married | **0.880** | 0.099 | 0.757; | 1.024 |  | | 0.891 | | | 0.130 | | | 0.768; | | 1.034 | | |  |
| widow/widower | 1.044 | 0.669 | 0.859; | 1.268 |  | | 1.039 | | | 0.677 | | | 0.867; | | 1.245 | | |  |
| divorced |  |  |  |  |  | |  | | |  | | |  | |  | | |  |
| residence area |  |  |  |  |  | |  | | |  | | |  | |  | | |  |
| urban | **1.145** | 0.012 | 1.031; | 1.273 |  | | **1.144** | | | 0.009 | | | 1.033; | | 1.266 | | |  |
| rural | **0.895** | 0.044 | 0.804; | 0.997 |  | | **0.895** | | | 0.034 | | | 0.808; | | 0.992 | | |  |
| moderate urban |  |  |  |  |  | |  | | |  | | |  | |  | | |  |
| **Enabling factors** |  |  |  |  |  | |  | | |  | | |  | |  | | |  |
| Income (quintiles) |  |  |  |  |  | |  | | |  | | |  | |  | | |  |
| Q1 (poorest) |  |  |  |  |  | |  | | |  | | |  | |  | | |  |
| Q2 | 1.145 | 0.099 | 0.975; | 1.345 |  | | 1.116 | | | 0.171 | | | 0.954; | | 1.305 | | |  |
| Q3 | 1.115 | 0.204 | 0.943; | 1.320 |  | | **1.151** | | | 0.093 | | | 0.977; | | 1.356 | | |  |
| Q4 | **1.344** | 0.001 | 1.137; | 1.589 |  | | **1.372** | | | 0.000 | | | 1.164; | | 1.616 | | |  |
| Q5 (richest) | **1.690** | 0.000 | 1.422; | 2.010 |  | | **1.715** | | | 0.000 | | | 1.448; | | 2.032 | | |  |
| employment_status |  |  |  |  |  | |  | | |  | | |  | |  | | |  |
| employed | 1.131 | 0.220 | 0.929; | 1.377 |  | | 1.136 | | | 0.196 | | | 0.937; | | 1.377 | | |  |
| unemployed | 1.131 | 0.306 | 0.894; | 1.430 |  | | 1.135 | | | 0.284 | | | 0.901; | | 1.430 | | |  |
| student | 1.265 | 0.158 | 0.913; | 1.754 |  | | 1.235 | | | 0.198 | | | 0.896; | | 1.704 | | |  |
| retired | 1.010 | 0.922 | 0.824; | 1.239 |  | | 1.005 | | | 0.957 | | | 0.827; | | 1.222 | | |  |
| all remaing status |  |  |  |  |  | |  | | |  | | |  | |  | | |  |
| financial_availability | **1.164** | 0.006 | 1.045; | 1.296 |  | | **1.152** | | | 0.007 | | | 1.040; | | 1.276 | | |  |
| **Needs** |  |  |  |  |  | |  | | |  | | |  | |  | | |  |
| SAH | **1.088** | 0.013 | 1.018; | 1.163 |  | | **1.105** | | | 0.002 | | | 1.037; | | 1.178 | | |  |
| chronic | 1.008 | 0.884 | 0.906; | 1.122 |  | | 1.007 | | | 0.899 | | | 0.907; | | 1.118 | | |  |
| pain intensity | **1.215** | 0.000 | 1.175; | 1.255 |  | | **1.172** | | | 0.000 | | | 1.135; | | 1.210 | | |  |
| lack sleep | **1.143** | 0.059 | 0.995; | 1.314 |  | |  | | |  | | |  | |  | | |  |
| lack courage | 0.922 | 0.432 | 0.753; | 1.129 |  | |  | | |  | | |  | |  | | |  |
| lack energy | 1.139 | 0.121 | 0.966; | 1.344 |  | |  | | |  | | |  | |  | | |  |
| lack focus | 0.883 | 0.347 | 0.681; | 1.145 |  | |  | | |  | | |  | |  | | |  |
| depression_score |  |  |  |  |  | | **1.036** | | | 0.000 | | | 1.024; | | 1.049 | | |  |
| **Unmet health care needs** |  |  |  |  |  | |  | | |  | | |  | |  | | |  |
| unmet_needs | **1.257** | 0.003 | 1.080; | 1.464 |  | | **1.190** | | | 0.020 | | | 1.027; | | 1.379 | | |  |
| waiting_list | **1.286** | 0.000 | 1.158; | 1.428 |  | | **1.248** | | | 0.000 | | | 1.128; | | 1.380 | | |  |
| waiting_distance | **0.757** | 0.018 | 0.601; | 0.953 |  | | **0.695** | | | 0.001 | | | 0.559; | | 0.865 | | |  |
| **Other Needs** |  |  |  |  |  | |  | | |  | | |  | |  | | |  |
| BMI | **0.988** | 0.020 | 0.978; | 0.998 |  | | **0.987** | | | 0.008 | | | 0.978; | | 0.997 | | |  |
| constant | **0.162** | 0.000 | 0.091; | 0.288 |  | | **0.133** | | | 0.000 | | | 0.075; | | 0.235 | | |  |
|  |  |  |  |  |  | |  | | |  | | |  | |  | | |  |
| Number of obs | 11,819 |  |  |  |  | | 13,142 | | |  | | |  | |  | | |  |
| Wald chi2(29) | 462.85 |  |  |  |  | | 490.84 | | |  | | |  | |  | | |  |
| Prob > chi2 | 0.00 |  |  |  |  | | 0.00 | | |  | | |  | |  | | |  |
| Pseudo R2 | 0.037 |  |  |  |  | | 0.036 | | |  | | |  | |  | | |  |
| Log pseudolikelihood | -6,174.491 | |  | | |  | -6,788.933 | | | | |  | |  | | |  |  |
|  |  |  |  | | |  |  | |  | | |  | |  | | |  |  |
| Note: _cons estimates baseline odds. | | | | | |  |  | |  | | |  | |  | | |  |  |
